# Network topology and intrinsic excitability of the existing network drive integration patterns in a model of adult neurogenesis

**DOI:** 10.1186/1471-2202-14-S1-P342

**Published:** 2013-07-08

**Authors:** James P Roach, Michal R Zochowski, Leonard M Sander

**Affiliations:** 1 Neuroscience Graduate Program, University of Michigan, Ann Arbor, MI, 48109, USA; 2 Department of Physics, University of Michigan, Ann Arbor, MI, 48109, USA; 3 Biophysics Program, University of Michigan, Ann Arbor, MI, 48109, USA

## 

Neurogenesis in the adult hippocampus is critical process in learning and memory where immature granule cells project dendrites to the molecular layer and axons to CA3 pyramidal cells[1]. While the developmental processes that govern the maturation of new granule cells have been well characterized, the manner in which the structure of the established network and the intrinsic excitability of the neurons within that network govern the incorporation of new neurons into the network is still an unanswered question. The formation of inward and outward connections is the most obvious characteristic of integration of maturing neurons. Thus expanding the understanding of how shifts the timing of a postsynaptic spike after presynaptic spikes, which is measured by the phase response curve (PRC), changes the wiring of new cells into the network is an important line of research. Our group has previously used a leaky integrate and fire (LIF) computational model to investigate how new neurons integrate into networks of various topologies and activity levels. A limitation of this study is that the PRC of LIF model neurons is type 1, or purely excitatory, while the PRC hippocampal CA3 cells have been shown to be type 2, or both excitatory and inhibitory[2]. To address this, we will perform simulations of networks using Hodgkin-Huxley model neurons, which have a PRC that can be switched from type 2 to type 1 by decreasing the conductance of a M-type slow voltage dependant potassium current[3]. The networks are laid upon a 2D lattice with external stimulation at the centermost cells. Networks composed of type 2 neurons display bursting dynamics with broad activity, while those composed of type 1 neurons have activity clustered around the stimulus and low levels of synchrony. These differences in network dynamics will change how newborn neurons form connections with the existing network. Using a factorial experimental design the incorporation newborn cells will be investigated in four ways; type 1 cells added to a type 1 network, type 1 cells added to a type 2 network, type 2 cells added to a type 1 network, and type 2 cells added to a type 2 network. When both new and mature cells are type 1, 24% of new cells reached an activity level high enough for survival. In the case of type 2 cells, all introduced survived. The results of these simulations for both PRC types will allow for comparison with our previous work and show how changes in neuronal excitability.
